# Ron Knockdown and Ron Monoclonal Antibody IMC-RON8 Sensitize Pancreatic Cancer to Histone Deacetylase Inhibitors (HDACi)

**DOI:** 10.1371/journal.pone.0069992

**Published:** 2013-07-29

**Authors:** Yi Zou, Gillian M. Howell, Lisa E. Humphrey, Jing Wang, Michael G. Brattain

**Affiliations:** Eppley Institute for Research in Cancer and Allied Diseases, University of Nebraska Medical Center, Omaha, Nebraska, United States of America; University of Texas Health Science Center, United States of America

## Abstract

Recepteur d’origine nantais (Ron) is overexpressed in a panel of pancreatic cancer cells and tissue samples from pancreatic cancer patients. Ron can be activated by its ligand macrophage stimulating protein (MSP), thereby activating oncogenic signaling pathways. Crosstalk between Ron and EGFR, c-Met, or IGF-1R may provide a mechanism underlying drug resistance. Thus, targeting Ron may represent a novel therapeutic strategy. IMC-RON8 is the first Ron monoclonal antibody (mAb) entering clinical trial for targeting Ron overexpression. Our studies show IMC-RON8 downmodulated Ron expression in pancreatic cancer cells and significantly blocked MSP-stimulated Ron activation, downstream Akt and ERK phosphorylation, and survivin mRNA expression. IMC-RON8 hindered MSP-induced cell migration and reduced cell transformation. Histone deacetylase inhibitors (HDACi) are reported to target expression of various genes through modification of nucleosome histones and non-histone proteins. Our work shows HDACi TSA and Panobinostat (PS) decreased Ron mRNA and protein expression in pancreatic cancer cells. PS also reduced downstream signaling of pAkt, survivin, and XIAP, as well as enhanced cell apoptosis. Interestingly, PS reduced colony formation in Ron knockdown cells to a greater extent than Ron scramble control cells in colony formation and soft agarose assays. IMC-RON8 could also sensitize pancreatic cancer cells to PS, as reflected by reduced colony numbers and size in combination treatment with IMC-RON8 and PS compared to single treatment alone. The co-treatment further reduced Ron expression and pAkt, and increased PARP cleavage compared to either treatment alone. This study suggests the potential for a novel combination approach which may ultimately be of value in treatment of pancreatic cancer.

## Introduction

Pancreatic cancer is a highly malignant disease, with approximately 40,000 new cases diagnosed in the US in 2012 [1]. The five-year survival rate is very low (<5%) [2]. Currently, less than 10% of patients are eligible for curative surgery, while more than 90% with locally advanced or metastatic diseases are treated with radiotherapy and/or chemotherapy [3]. Pancreatic cancer eventually develops resistance to these therapies. Molecular studies revealed that genetic and epigenetic changes drive pancreatic cancer [4]. A better understanding of the molecular basis of pancreatic cancer will benefit development of novel therapeutic strategies.

Recently, Ron has been identified to be overexpressed in a subset of pancreatic cancer patients and established cancer cell lines [5], [6]. Ron belongs to MET receptor tyrosine kinase (RTK) family. Previous studies showed that Ron levels are elevated in many epithelial cancers including breast [7], colon [8], lung [9], and bladder [10] cancers. Ron overexpression was prognostic of poor survival and correlated with disease progression [11].

Functional studies showed that Ron can be activated by its ligand MSP to initiate a cascade of molecular signaling, including PI3K/Akt, MAPK, β-catenin, JNK and FAK pathways to regulate various cellular functions [12]. The MSP/Ron axis has been shown to influence cell migration and invasion, and potentially promote tumor metastasis [12], [13]. Downregulation of Ron by knockdown resulted in reduced cell proliferation, transformation, tumor growth, metastasis and increased cell apoptosis, in colon cancer cells [14], [15]; and sensitized pancreatic cancer cells to gemcitabine [16]. Therefore, Ron plays an important role in maintaining malignant phenotypes in human cancers. IMC-41A10 was the only human anti-Ron mAb that has been reported to have anticancer activity [17]. IMC-41A10 inhibited MSP binding to Ron, reduced MSP-mediated Ron phosphorylation, PI3K/Akt and MAPK activation, and cell migration *in vitro*. Recently, a novel human mAb IMC-RON8 was generated and entered phase I clinical trial as the first Ron mAb.

Drug resistance is a major hurdle in cancer therapeutics. Crosstalk between RTKs may represent one of the mechanisms for drug resistance. Ron has been reported to hetero-dimerize with c-Met [18], and cross-talk with EGFR [19], [20] to activate the RTKs in a reciprocal manner. Integrin and IGF1-R can also activate Ron through an MSP-independent manner [21], [22]. Overexpression of Ron increased drug resistance to IGF1-R inhibitor BMS-536924 in childhood sarcoma [23]; while knockdown of Ron sensitized the resistant cells to BMS-536924 [23]. These findings underscore a potential benefit for rational combination treatments based on Ron tyrosine kinase networks.

Epigenetic changes, such as acetylation and methylation, are the key post-translational modifications of the highly charged lysine residues of core nucleosomal histones. Changes to histone acetylation are under the control of opposing activities of histone acetytransferases (HATs) and histone deacetylases (HDACs) [24], which determine the transcriptional activation or repression of gene expression. HATs and HDACs also play an important role in acetylation and deacetylation of non-histone proteins [25], [26].

Overexpression of HDACs has been found in colon, breast, pancreatic, and other cancers [27]–[31], which suggest that they may be attractive anticancer targets. HDAC inhibitors are reported to target expression of various genes through modification of nucleosome histones and non-histone proteins. The pan-HDACi TSA, Vorinostat, Belinostat and Panobinostat (PS) have been widely investigated. PS exerted robust anticancer effects in leukemia cells through hyperacetylation of nucleosome histones and transcriptional upregulation of p21 and p27 expression [32]. PS reduced pancreatic cancer growth through inhibition of cell proliferation and induction of cell apoptosis [33]. TSA enhanced the response of pancreatic cancer cells to conventional chemotherapies including 5-FU and gemcitabine [2], [34]. However, the precise mechanisms underlying HDACi treatment are largely unknown. Our lab showed that HDACi Belinostat induced the expression of epigenetically silent TGFβ RII and therefore restored TGFβ signaling-mediated cell growth inhibition in Smad4 wild type (wt) pancreatic cancer cells (e.g., Miapaca-2), [35]. Ron is highly expressed in Smad4 mutant pancreatic cancer cells. Wild type Smad4 expression was reported to suppress Ron expression [36]. Knockdown of Smad4 or inhibition of TGFβ signaling resulted in induction of Ron expression. The studies here show that HDACi PS and TSA downregulated Ron and its downstream signaling in pancreatic cancer cells. Ron KD or Ron mAb sensitized pancreatic cancer cells to PS. Oral PS has been used in phase II clinical trial in adult patients with refractory/relapsed Hodgkin’s lymphoma and phase I trial for advanced solid tumor [32]. Our studies have explored a possible mechanism underlying PS treatment of pancreatic cancer and provided important evidence for the potential of a rational combination treatment for Ron-expressing pancreatic cancer cells.

## Materials and Methods

### Cell Cultures

Human pancreatic cancer cell lines Capan-1 and CFPAC-1 were obtained from the American Type Culture Collection (ATCC, Rockville, MD), and L3.6pl cells were originated from Dr. I. J. Fidler [37]. Cells were cultured in SM medium (McCoy’s 5A serum-free medium with pyruvate, vitamins, amino acids and antibiotics), supplemented with 10% FBS. All cells were maintained in a humidified atmosphere and 5% CO_2_ at 37°C.

### Antibodies and Reagents

Antibodies for Ron C-20, survivin, MAPK and pERK1/2 were purchased from Santa Cruz Biotechnology Inc. Poly-(ADP-ribose) polymerase (PARP), pAkt (Ser^473^), Akt, Caspase 9 antibodies were obtained from Cell Signaling Technology. XIAP antibody was from Abcam. Anti-pTry clone 4G10 was purchased from Millipore. Antibody for actin was purchased from Sigma.

Recombinant human MSP was purchased from R&D systems (Minneapolis, MN) and used at a concentration of 400ng/ml in all experiments. IMC-RON8 was provided by ImClone LLC system (New York, NY). Panobinostat (PS) was obtained from Selleckchem. Trichostatin (TSA) was from Sigma-Aldrich. Both PS and TSA were solubilised in DMSO and stored at −80°C until use.

### Generation of Ron Knockdown Stable Cell Lines

The scramble (pSR-SC) and shRNA pSR-Ron expression plasmids were provided by Dr. J. Wang [15]. To establish stable cell lines with Ron knocked down by shRNA for L3.6pl cells, pSR-Ron and a control vector (pSR-SC) were co-transfected into a HEK293GP package cell line together with vesicular stomatitis virus-G (pVSV-G) plasmid using Fugene HD transfection reagent. The supernatant was collected 48 h later and used to infect L3.6pl cells. Puromycin was used to select infected cells. Individual cells were then isolated by plating the pooled cells at low densities. Two clones A6 and B21 with Ron specific KD were used in the further studies.

### Western Blot Analysis

Cells were lysed in NP40 lysis buffer [50 mmol/L Tris-HCl (pH 7.4), 150 mmol/L NaCl, 0.5% NP40, 50 mmol/L NaF, 1 mmol/L Na_3_VO_3_, 1 mmol/L phenylmethylsulfonyl fluoride, 1 mmol/L DTT, 25 µg/mL aprotinin, 25 µg/mL trypsin inhibitor, and 25 µg/mL leupeptin] at 4°C. The cell lysates were boiled in a gel-loading buffer and separated by SDS/PAGE in 4∼15% gels. After PAGE, the proteins were transferred to a PVDF membrane (Millipore). Following transfer, the membranes were blocked with 5% milk in TBST for 1 hour. Then the membranes were probed with the indicated primary antibodies at 4°C overnight. After washing three times in TBST, the membranes were incubated for 1 hour with the appropriate species-specific HRP-conjugated secondary antibodies. After another three-time wash, the membranes were processed and visualized with enhanced chemiluminescence (ECL) reagents (Amersham).

### *In Vitro* Proliferation Assay Using MTT

Cell proliferation was analyzed using 3-(4,5-dimethylthiazol-2-yl)-2,5- diphenyltetrazolium bromide (MTT) assay. Briefly, Capan-1, CFPAC-1 and L3.6pl cells were seeded at a density of 2000∼3000 cells/well in 96-well plates. The cells were treated with different concentrations of PS on day 2. Forty-eight hours following treatment, the cells were then incubated with MTT (0.5mg/ml) for 2 hours at 37°C. After the medium containing MTT was removed, 150µl of DMSO were added to each well and mixed on the rocker. The plates were read at 570 nm using a microplate reader (Bio-Rad). The absorbance measured is directly proportional to the number of the viable cells in the culture.

### DNA Fragmentation (Cell death ELISA)

Apoptosis was quantified using the DNA fragmentation Cell Death Detection ELISA Plus kit (Roche) according to the manufacturer’s instructions. Cells were treated with PS as described above. Fold increases of DNA fragmentation were normalized with MTT values from identical treatment conditions.

### RNA Extraction and Quantitative Real-time RT-PCR

Total RNA was prepared from treated cells using the High Pure RNA isolation kit (Roche). Expression of Ron mRNA was measured by quantitative real-time PCR with TaqMan reagents (Applied Biosystems) on cDNAs reverse transcribed from 2 µg total RNA. The GAPDH mRNA was amplified simultaneously for an endogenous control.

### Immunoprecipitation (IP)

Cell lysates with 600 µg protein were incubated with 10 µg anti-Ron antibody and Sepharose beads overnight at 4°C. The following day, the beads were collected by centrifuge and the supernatant was removed for further analysis. The beads were washed three times with PBST. After the final wash, the pelletted beads were resuspended in 30 µl of SDS sample buffer. The samples were heated at 95°C for 5 min. The resulting immune complexes were run on an 8% SDS-PAGE gel followed by Immunoblot (IB) for pTyr and Ron.

### Would Healing Assays and Transwell Motility Assays

Capan-1 cells were seeded in 60mm-dish and grew till confluence. They were then starved overnight before IMC-RON8 treatment for 1 h. Confluent cell monolayers were then manually wounded by a pipette tip to form a gap. The cell culture medium was replaced with fresh SM medium followed by MSP stimulation for 24h and 48h. Wound closure was monitored by microscopy.

Cell migration in response to MSP was measured utilizing a modified Boyden chamber with 8 µm pore 6.5-mm polycarbonate transwell filters (Corning Costar). Following trypsinization, single cell suspensions were seeded onto the upper chambers in SM medium in 24-well plates. Chemo-attractant MSP was added in the bottom chambers, with 10% FBS was used as a positive control. IMC-RON8 was added in the upper chamber 2 hours before the addition of MSP. Cells were allowed to migrate toward bottom chamber with chemo-attractants. After 24h incubation, 3-(4,5-dimethylthiazol-2-yl)-2,5-diphenyltetrazolium bromide was added to the medium. The cells on the upper chamber of the filter were removed with a cotton swab, and the cells migrating to the underside of the filter were visualized under the microscope followed by solubilization in DMSO and quantification at 570 nm.

### Colony Formation Assays and Soft Agarose Assays

L3.6pl SC cells and shRNA KD clones A6 and B21 were plated in 24-well plates at the density of 300 cells/well. Cells were treated with PS on day 2 at indicated concentrations for 48 hours. Then the cells were washed with PBS three times. Fresh media with 10%FBS were added. After an additional 10 days of incubation, cell colonies were visualized by staining with MTT, followed by solubilization in DMSO and quatifying at 570nM.

Soft agarose assays were used to determine the transformation properties of CFPAC-1 and L3.6pl cells in the presence or absence of IMC-RON8 and/or PS. Briefly, the cells were suspended at 2000 cells/ml in 1 ml of 0.4% agarose in SM medium containing 10% FBS and plated on top of 1 ml of 0.8% agarose in the same medium in 6-well plates. Plates were incubated for 2 weeks at 37°C with 5% CO_2_ in a humidified incubator. Cell colonies were visualized by staining with 0.5 mol of *p*-iodonitrotetrazolium violet (Sigma) and photographed. Colony numbers were counted. The anchorage-independent growth of L3.6pl SC and Ron KD cells were also measured by soft agarose assays with or without PS treatment.

### Statistical Analysis

Data were summarized as mean ± standard error (S.E.). The statistical significance was calculated using SPSS software with student *t* test or ANOVA to determine the differences in means. The significance was accepted for *p* value *<*0.05. All experiments were repeated three times independently.

## Results

### IMC-RON8 Downmodulated Ron Expression and Inhibited MSP-dependent Ron Phosphorylation and Downstream Signaling

**Figure 1:**
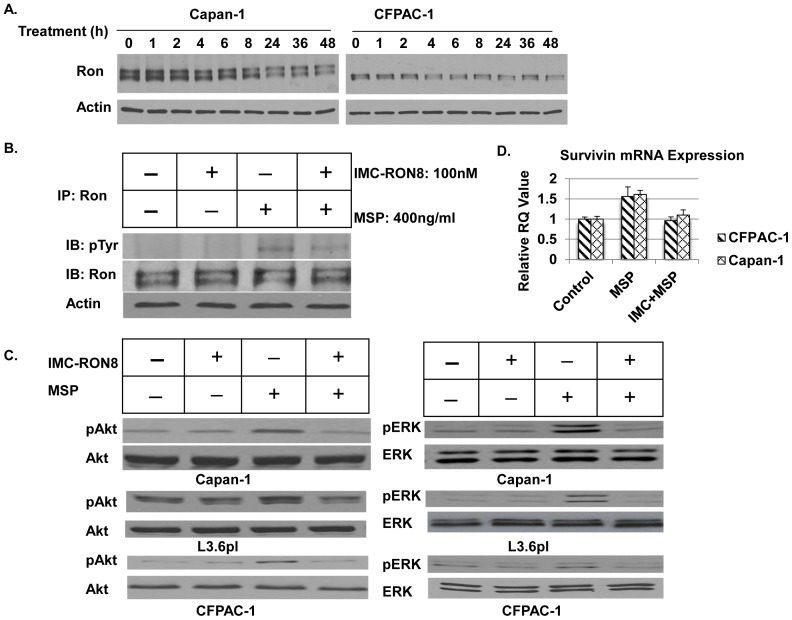
IMC-RON8 downmodulated Ron expression and inhibited MSP-dependent Ron phosphorylation and downstream signaling in pancreatic cancer cells. A) Effect of IMC-RON8 (100nM) on Ron expression. Western blot for Ron. β-actin served as a loading control. B) Effect of IMC-RON8 on MSP-induced Ron activation. Cells were serum starved overnight and treated with IMC-RON8 for 1 hour followed by MSP stimulation for half hour. Cell lysates were subjected to IP, and IB for pRon. C) Effect of IMC-RON8 on downstream signaling. Same treatment as B). Western blot for pERK and pAkt. D) Ron activation and survivin mRNA expression. Capan-1 and CFPAC-1 cells were treated with IMC-RON8 followed by MSP stimulation for 12 hours after serum starvation for overnight. RT- qPCR was performed on total RNA to determine mRNA level of survivin. GAPDH mRNA was used as an internal control.

To evaluate the effect of IMC-RON8 on Ron expression in Ron-overexpressing pancreatic cancer cells, Capan-1 and CFPAC-1 cells were treated with 100nM of IMC-RON8 for various time intervals followed by western blot analysis. Figure 1A showed that Ron reduction was seen after 4h treatment with IMC-RON8 and lasted up to 48 hours in both cell lines. IMC-RON8 was then evaluated for its ability to block Ron activation and its downstream effectors, MAPK and Akt. CFPAC-1 cells were serum starved overnight and treated with IMC-RON8 followed by MSP stimulation for 30 minutes. Results from IP (Fig. 1B) demonstrated that IMC-RON8 itself did not induce Ron phosphorylation, indicating that IMC-RON8 did not have agonist effects. Tyrosine phosphorylation of Ron was significantly increased by MSP stimulation, but largely abrogated by IMC-RON8 treatment. A previous study reported that MSP stimulation activates Ron downstream signaling PI3K/Akt and MAPK in pancreatic cancer cells [6]. Consequently, we investigated the role of IMC-RON8 on MAPK and Akt phosphorylation in Capan-1, L3.6pl and CFPAC-1 cells. Our results showed that all the pancreatic cancer cell lines we examined exhibited a strong MSP-dependent phosphorylation of Akt and MAPK, and that IMC-RON8 treatment blocked MSP-induced Akt and MAPK activation in all the cell lines (Fig. 1C). To determine whether Ron activation may increase survivin and XIAP expression, we treated Capan-1 and CFPAC-1 cells with IMC-RON8 followed by MSP stimulation for 12 hours after serum starvation for overnight. The results showed that MSP stimulation increased survivin mRNA expression (Fig. 1D), but not XIAP mRNA level (data not shown). IMC-RON8 can block this increased survivin mRNA expression. We did not observe significant increase of XIAP and survivin protein expression after MSP stimulation on western blotting.

### IMC-RON8 Significantly Inhibited MSP-driven Cell Motility

**Figure 2:**
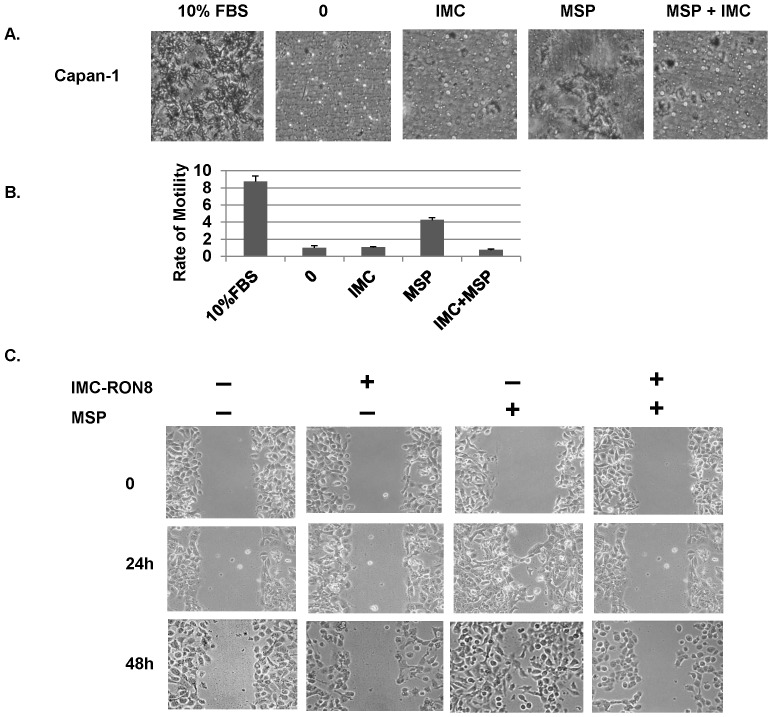
Effects of IMC-RON8 on MSP-mediated cell migration. Capan-1 cells were serum starved overnight. Cell migration was assessed by transwell assay with MSP as a chemoattractant in the bottom chamber. A) Images of cells successfully migrated to the bottom side of the transwell membrane. B) Quantification of migrated cells. C. *In vitro* wound healing assay.

To assess the effect of IMC-RON8 on cell motility, a transwell migration assay was performed in Capan-1 cells with IMC-RON8 treatment in the presence or absence of MSP. IMC-RON8 significantly reduced MSP- induced cell migration in Capan-1 cells by 80∼90% (Fig. 2A). *In vitro* wound healing assays further confirmed the ability of IMC-RON8 to block cell migration. Capan-1 cells stimulated with MSP started to migrate to the wound area 24 hours after the scratch (Fig. 2B). The wound was nearly healed by migrating cells at 48 hours after MSP treatment, while IMC-RON8 significantly reduced cell mobility.

### HDACi Downregulated Ron mRNA and Protein Expression and Downstream Signaling

**Figure 3:**
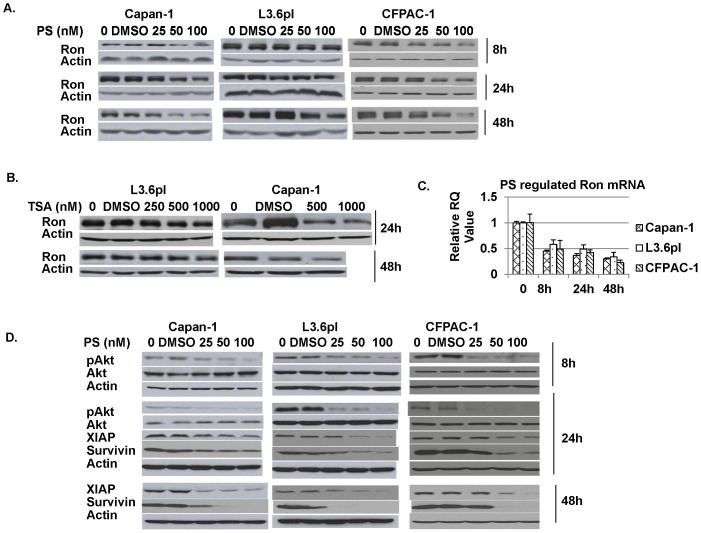
Panobinostat downregulated Ron mRNA, protein expression and decreased downstream signaling through pAkt, XIAP, and survivin. A) Pancreatic cancer cells were treated with different concentrations of PS for 8h, 24h, and 48 hours. Western blot for Ron was performed. β actin served as a loading control. B) Cells were treated with TSA with IB for Ron. Substantial number of cells were dead (about 80%) in 1000nM TSA-treated Capan-1 cells at 48 hours. C) Cells were treated with 50nM of PS for 8, 24 and 48 hours. RT- qPCR was performed on total RNA to determine mRNA level of Ron. GAPDH mRNA was used as an internal control. D) Cells were treated with different concentrations of PS at the indicated time points. Western blot for pAkt, XIAP and survivin expression. B-actin was used a loading control.

To determine the effect of HDACi on Ron expression, Capan-1, L3.6pl and CFPAC-1 cells were treated with pan-HDACi PS for 8, 24 and 48 hours. The results showed that PS depleted Ron expression in all Ron-expressing cell lines examined (Fig. 3A). Reduction of Ron was noted within 8 hours with increasing depletion occurring over 48 h periods (Fig. 3A). The pronounced downregulation of Ron was also seen in pancreatic cancer cells treated with another pan-HDACi, TSA (Fig. 3B), indicating Ron reduction is a common feature for Pan-HDACi treatment. We then determined whether changes in Ron protein were at least partially due to changes in its transcription. Treatment with PS significantly decreased the mRNA level of Ron in a time-dependent manner (Fig. 3C). In addition, the cells treated with DMSO alone (solvent for PS and TSA) had no influence on Ron expression both at the mRNA and protein levels. Previous studies showed that Ron phosphorylation activates a cascade of downstream signaling molecules including Akt phosphorylation [6], [15], [17]. We next determined the effects of PS on downstream cell signaling. Capan-1, L3.6pl and CFPAC-1 cells were treated with PS. After treatment for 8 and 24 hours, Akt phosphorylation was reduced in a concentration-dependent manner in all 3 pancreatic cancer cell lines (Fig. 3D). PS treatment for 24 and 48 hours also reduced both XIAP and survivin protein levels in a concentration-dependent manner in pancreatic cancer cells (Fig. 3D).

### PS Reduced Cell Growth and Increased Cell Apoptosis Through Induction of Cell Cycle Regulator p21 and Caspase Activity

**Figure 4:**
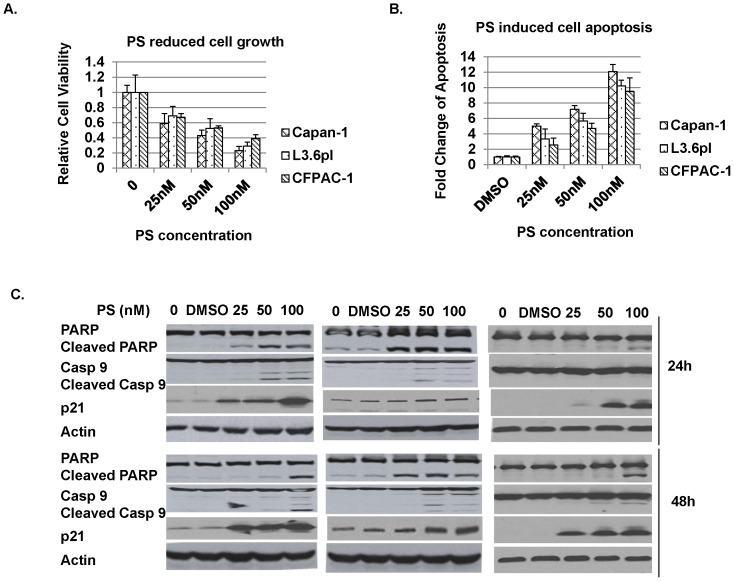
Panobinostat reduced cell growth and increased apoptosis through induction of cell cycle regulator p21 and caspase activity, respectively. Cells were treated with different concentrations of PS, A) cell growth was measured by MTT; B) cell apoptosis was measured by DNA fragmentation; C) Western blots for p21, and caspase activity (PARP and caspase 9 cleavages). B-actin was used as a loading control.

The function of IAP family members XIAP and survivin is to inhibit caspase 3, 7, 9 activity and caspase-dependent cell apoptosis by binding to the active caspases [42]. We show that after 48h of incubation, PS reduced cell proliferation (Fig. 4A) and increased cell apoptosis (Fig. 4B) in a concentration-dependent manner in all 3 cell lines tested as determined by MTT and DNA fragmentation. Previous studies reported that HDACi treatment results in impaired proliferation of pancreatic cancer cells due to an arrest in cell cycle and the induction of the caspase-dependent programmed cell death [33]. We detected an increase of the cyclin-dependent kinase inhibitor (CDK) p21 in pancreatic cancer cells after HDACi treatment (Fig. 4C). Increased Capase-9 and PARP cleavage following caspase activation was also observed in Capan-1 and L3.6pl cells (Fig. 4C). We found that in CFPAC-1 cells, increased caspase 9 cleavage was only detected at high concentrations of PS treatment at 48 hours, but not at 24 hours. Correspondingly, PARP cleavage was seen only at the high concentration of PS treatment in CFPAC-1 cells, thus indicating that CFPAC-1 cells are relatively resistant to PS induced-cell apoptosis compared to Capan-1 and L3.6pl cells.

### Ron Knockdown or Ron mAb IMC-RON8 Sensitized Pancreatic Cancer Cells to PS Treatment

**Figure 5:**
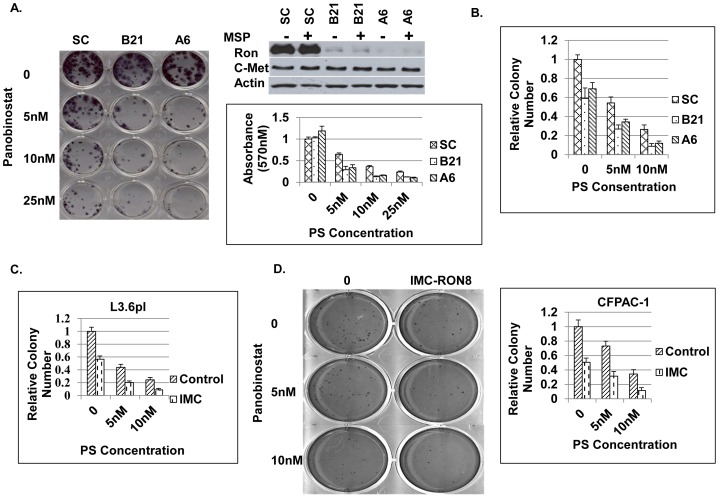
Ron knockdown or Ron mAb IMC-RON8 sensitized pancreatic cancer cells to Panobinostat. A) L3.6pl cells were stably transfected with Ron SC and shRNA plasmids. Ron KD clones A6 and B21 were selected for clonogenic assay: L3.6pl Ron SC and KD clones A6 and B21 were treated on day 2 with PS at indicated concentrations for 48 hours. After washing, cells were cultured for an additional 10 days in 10% FBS containing medium. Cell colonies were visualized after MTT staining and quantified in DMSO. B). Anchorage-independent growth of L3.6pl SC and Ron KD clones treated by PS was determined as described in Materials and Methods. Soft argrose assay was also performed for C) L3.6pl cells and D) CFPAC-1 cells treated with either IMC-RON8 or PS alone or their combinations.

Both IMC-RON8 and PS downmodulated Ron expression. IMC-RON8 reduces oncogenic signaling through pAkt and pERK and inhibits cell migration in pancreatic cancer cells. However, IMC-RON8 does not affect cell proliferation and cell apoptosis *in vitro* as evidenced by MTT, PARP and caspase 9 cleavages, which is the same for Ron knockdown (data not shown). PS, however, significantly induces cell apoptosis and reduces cell proliferation in pancreatic cancer cells. The combination between Ron knockdown or IMC-RON8 and PS may compensate for the lack of inhibition of cell survival in Ron targeting IMC-RON8 or Ron knockdown. To determine the proliferative effects of Ron knockdown and PS treatment, a standard colony formation assay was performed. First, the scrambled (SC) and Ron shRNA plasmids were delivered into L3.6pl cells by transfection-infection to establish L3.6pl SC control and Ron knockdown stable cell lines. Single cell clones A6 and B21 were selected after puromycin treatment. Ron shRNA specifically knocked down Ron expression, since c-Met which has high sequence identity with Ron, did not show any change (Fig. 5A). Then L3.6pl SC and Ron KD clones A6 and B21 cells were treated with low concentrations of PS. The results showed that both L3.6pl SC and Ron KD cell growth were significantly inhibited by PS in a concentration-dependent manner (Fig. 5A). PS treatment at the same concentration reduced colony formation in Ron KD L3.6pl cells to a greater extent than Ron SC control cells (Fig. 5A). Ron KD itself did not change cell proliferation in pancreatic cancer cells. We next conducted soft agarose assays to further determine the transformation properties of L3.6pl cells with Ron KD and PS treatment. Figure 5B showed that L3.6pl Ron KD clones produced fewer colonies (30∼40%) than Ron SC cells in the soft agarose. PS significantly reduced colonies formed in both L3.6pl Ron SC and KD cell clones (Fig. 5B). Interestingly, PS treatment led to significantly further fewer colony numbers in L3.6pl Ron KD cells than in L3.6pl SC cells at all PS concentrations (Fig. 5B). The sizes of colonies in PS treated L3.6pl Ron KD cells were obviously smaller than PS treated SC cells (data not shown). To further determine the role of Ron in pancreatic cancer, IMC-RON8 was introduced to measure potential combination effects together with PS. In both L3.6pl (Fig. 5C) and CFPAC-1 (Fig. 5D) cells, blockade of Ron with IMC-RON8 could reduce colony formation compared to the controls without any treatment (up to 50%). Combination treatment with PS and IMC-RON8 further generated significantly fewer colony numbers with smaller size than single drug treatment alone in both L3.6pl and CFPAC-1 cells (Fig. 5C and 5D).

### The Mechanisms Underlie IMC-RON8 Sensitization of Pancreatic Cancer Cells to PS

**Figure 6:**
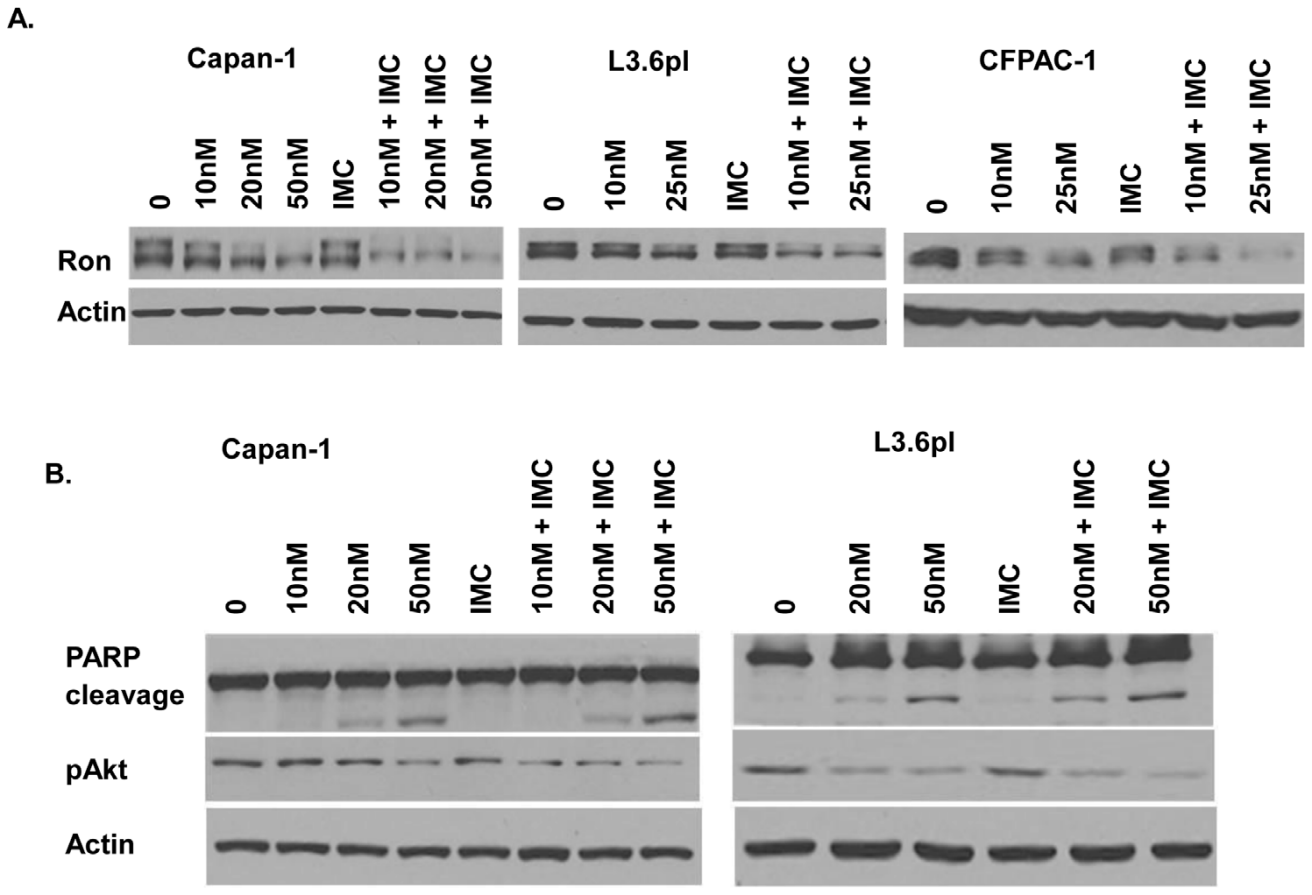
Mechanisms underlie IMC-RON8 sensitization of pancreatic cancer cells to Panobinostat. A) Ron expression after co-treatment of Capan-1, L3.6pl and CFPAC-1 cells with IMC-RON8 and PS. B) Effect of co-treatment of IMC-RON8 and PS on pAkt and PARP cleavage. β-actin was used as a loading control.

To determine the effect of combination treatment with PS and IMC-RON8 on Ron expression, Capan-1, L3.6pl and CFPAC-1 cells were treated with the indicated concentrations of IMC-RON8, PS or their combination. We found that Ron was degraded by IMC-RON8 after 24h treatment (Fig. 6A). PS single treatment reduced Ron, pAkt and increased PARP cleavage. However, co-treatment further lowered Ron expression (Fig. 6A), compared to single treatment alone in all three pancreatic cancer cell lines we examined. Moreover, co-treatment also further reduced pAkt and increased PARP cleavage (Fig 6B). These might explain the reduced colony formation in combination treatment relative to single agent alone.

## Discussion

This study identified a potential novel therapeutic approach in pancreatic cancer using a combination strategy through exploiting both genetic and epigenetic features. Pancreatic cancer is one of the most challenging problems in cancer therapy. Current chemotherapy by gemcitabine has a very low response rate (<20%) and drug resistance develops rapidly resulting in treatment failure [2]. Thus, new therapeutic strategies are urgently needed.

Ron has been recently reported to be highly expressed in pancreatic cancer cells and patient samples [5], [6]. Stimulation with MSP activates Ron and its downstream signaling, including PI3K/Akt and MAPK and promotes cell migration and invasion. However, Ron activation had no effect on proliferation in pancreatic cancer cells [6]. Knockdown of Ron has shown increased susceptibility to apoptosis of colon cancer cells to growth factor deprivation stress through mutant p110α activation [14], [15]. However, pancreatic cancer cells do not contain p110α mutations. Ron KD had no effect on cell proliferation and apoptosis as assessed by MTT, PARP and caspase 9 cleavages *in vitro* (data not shown) in pancreatic cancer cells.

Our studies here showed that IMC-RON8 downmodulated Ron expression, which was consistent with previous studies that mouse anti-Ron mAbs Zt/g4, Zt/f2 and Zt/c9 reduced Ron expression in colon cancer cells [43]. Human mAb IMC-41A10 efficiently reduced MSP-mediated Ron activation and its downstream PI3K/Akt and MAPK activation [17]. MAPK signaling reduction by IMC41A10 was evidenced by pERK reduction in all the cancer cell lines chosen. However, the effect of IMC41A10 on pAkt is not consistent in all the cell lines. For example, IMC41A10 had strong impact on the reduction of Akt activation in HT29, Du-145 and AGS, whereas IMC-41A10 did not change pAkt in other cells including the pancreatic cancer cell line BxPC3 [17].

IMC-RON8, another fully human anti-Ron mAb, displayed antitumor activity against human colon, lung and pancreatic xenografts in nude mice [44]. Our studies here demonstrated that IMC-RON8 effectively inhibited Ron phosphorylation in CFPAC-1 cells, as well as downstream pMAPK and pAkt activation in all the pancreatic cancer cell lines we examined including BxPC3 (data not shown). This indicated that IMC-RON8 is functional for inhibiting MSP-mediated signaling pathways and exhibits strong efficacy with respect to blocking the PI3K/Akt pathway.

Previous work from our lab and others has demonstrated that Akt activation is linked to members of the inhibitor of apoptosis (IAP) family such as XIAP and survivin, which are overexpressed and dysregulated in many human cancers [35], [38]–[41]. Akt phosphorylation of XIAP led to increased stability and decreased cell apoptosis in ovarian cancer treated with cisplatin [40]. The PI3K/Akt pathway mediated by many growth factors was reported to upregulate survivin expression [39], [41]. Our experiment found that MSP induced Ron activation increased survivin but not XIAP mRNA expression. The protein level did not significantly change.

Pancreatic cancer is a highly aggressive disease with a propensity for early invasion and metastasis. Ron is rarely expressed in normal pancreatic ducts or early pancreatic intraepithelial neoplasia (PanIN). The expression level of Ron is increased in invasive and metastatic cancer and correlates with tumor progression in pancreatic cancer patient samples [6]. Studies showed that MSP-mediated Ron activation significantly increased cell migration and invasion [14], [38]. The PI3K/Akt pathway is required for epithelial cell migration activated by MSP [45]. Substantial cell migration and invasion was also seen in pancreatic cancer with Ron-overexpression [5], [6] and was associated with EMT [5]. The effect of IMC-RON8 on Ron-mediated cell migration was evaluated in our studies by transwell and wound healing assays. IMC-RON8 strongly inhibited MSP-dependent cell migration in transwell assays. Wound healing assays showed that a robust healing response to MSP was blocked by IMC-RON8 before MSP stimulation. It is reasonable to postulate that IMC-RON8 treatment in pancreatic cancer may reduce the invasive and metastatic phenotype activated by circulating MSP.

The PI3K/Akt and MAPK signaling pathways have been reported to be involved in Ron-mediated anchorage independent growth in colon epithelial cells [14]. Ron KD resulted in reduced cell transformation in colon cancer cells [14], [15]. Although IMC-RON8 had no effects on cell proliferation and apoptosis as assessed by MTT, PARP and caspase 9 cleavage in pancreatic cancer cells (data not shown), anchorage independent growth was significantly impaired with IMC-RON8 treatment. The same reduction could also be seen in Ron KD L3.6pl cell clones, where Ron KD resulted in reduced colony formation compared to Ron SC cells.

HDACs play an important role in the epigenetic regulation of gene expression in human cancers, including pancreatic cancer [27], [28], [46]. Recently, development of HDAC inhibitors and their usage in combination therapy has emerged as a promising strategy.

The HDACi TSA, Vorinostat, Panobinostat and Belinostat have been a focus for recent cancer studies. TSA treatment of pancreatic cancer cells inhibited cell proliferation amd induced cell apoptosis through cell cycle arrest and altered expression of pro-apoptotic gene (BIM) versus anti-apoptotic genes (Bcl-xL and Bcl-W) [34], [47], [48]. Vorinostat was reported to induce growth inhibition in pancreatic cancer cell lines through p21 induction [49]. In 2008, two novel hydroxamic acids LAQ824 and PS were found to significantly suppress cell growth in seven p53 mutant pancreatic cancer cell lines through upregulation of p21 [50]. Our studies here also demonstrated that PS treatment of pancreatic cancer cells significantly reduced cell proliferation at nanomolar concentrations, and induced cell apoptosis. The mechanism underlying the HDACi effects on pancreatic cancer was investigated.

We showed that PS reduced Ron expression in Capan-1, CFPAC-1 and L3.6pl cells, and thereby decreased its downstream signaling, leading to inactivation of Akt. Previous studies reported that histone deacetylase inhibitor (HDACi) LAQ824 reduced EGFR and HER2 expression in breast cancer cells [51]. Our experiments also showed that HDACi Panobinostat (PS) reduced EGFR and c-Met expression in pancreatic cancer cells (data not shown). Since IMC-RON8 only blocked survivin (but not XIAP) mRNA expression. We postulate that HDAC inhibitor PS decreased XIAP and survivin expression may due to the combinational reduction of Ron, EGFR and c-Met. PS also induced caspase-dependent cell apoptosis as evidenced by increased PARP and caspase 9 cleavages.

Although the first human Ron mAb IMC-41A10 was not reported to downmodulate Ron expression, our studies found that IMC-RON8 treatment promoted Ron degradation in pancreatic cancer cells. Interestingly, combination of PS and IMC-RON8 further reduced Ron expression compared to each single treatment. This was associated with reduced colony formation by anchorage-independent growth assays in the combination group compared to individual agent alone in the pancreatic cancer cells examined. L3.6pl cells with Ron knockdown are more sensitive to PS as exhibited by fewer colony numbers in Ron KD cell clones A6 and B21 than in L3.6pl SC cells in both colony formation assays and soft agarose assays. We also determined PARP cleavage and pAkt by western blot, with PS and IMC-RON8 treatment alone or in combination. We found combination treatment seems further reduced pAkt and increased PARP cleavage compared to PS treatment alone. We did not see significant changes in XIAP and survivin expression. Our study provides evidence that combination treatment of PS and IMC-RON8 appears to have potential with regard to the treatment of pancreatic cancer due to Ron overexpression.
